# Survival improvement and prognosis for hepatocellular carcinoma: analysis of the SEER database

**DOI:** 10.1186/s12885-021-08904-3

**Published:** 2021-10-29

**Authors:** Jingli Ding, Zhili Wen

**Affiliations:** 1 Department of Gastroenterology, Zhongnan Hospital of Wuhan University, No. 169, Donghu Road, Hubei, Wuhan 430071 China; 2 Department of Gastroenterology, The Second Affiliated Hospital of Nanchang University, No. 1 Minde Road, Jiangxi, Nanchang 330006 China

**Keywords:** Liver cancer, Survival trend, Prognosis, Overall survival, Disease specific survival

## Abstract

**Background:**

Hepatocellular carcinoma (HCC) incidences have been increasing in the United States. This study aimed to examine temporal trend of HCC survival and determine prognostic factors influencing HCC survival within the U.S. population.

**Methods:**

The Surveillance Epidemiology, and End Results (SEER) database was used to identify patients diagnosed with primary HCC from 1988 to 2015. Overall survival (OS) and disease-specific survival (DSS) were calculated by the Kaplan-Meier method. Univariate and multivariate Cox regression models were used to estimate hazard ratios (HRs) and 95% confidence intervals (CIs) for prognostic factors and comparing survival between patients diagnosed at different periods (per 5-year interval).

**Results**

A total of 80,347 patients were included. The proportions of both young patients (< 45 years) and old patients (≥75 years) decreased over time (*P* < 0.001) and the male-to-female ratio increased over time (*P* < 0.001). Significant decreasing temporal trends were observed for HCC severity at diagnosis, including SEER stage, tumor size, tumor extent, and lymph node involvement (*P* < 0.001 for all). OS and DSS of patients with HCC improved over time (*P* < 0.001). After adjusting for patient and tumor characteristics and treatment difference, period of diagnosis retained an independent factor for improved DSS and its prognostic significance was evident for localized and regional HCC (*P* < 0.001), but not for distant HCC. On multivariate analyses, young age, female gender, Hispanic ethnicity, and married status were predictors favoring DSS, whereas a worse DSS was observed for patients with tumor > 5 cm, with vascular invasion, and with lymph node involvement. Patients treated with liver-directed therapy (HR = 0.54, 95% CI: 0.35–0.56), hepatic resection (HR = 0.35, 95% CI: 0.33–0.37), and transplantation (HR = 0.14, 95% CI: 0.13–0.15) had significantly longer DSS compared with those who received no surgery. In stratified analyses, the beneficial effects of surgical approach, regardless therapy type, were significant across all stages.

**Conclusions:**

Our results indicate a significant improvement in survival for HCC patients from 1988 to 2015, which may be attributable to advances in early diagnosis and therapeutic approaches.

## Background

Hepatocellular carcinoma (HCC) is the third leading cause of cancer-related mortality worldwide, with more than 780,000 deaths occurred in 2018 [1]. The United States is among the low-risk regions for HCC, but HCC incidence in the United States has more than tripled since 1980s, with the rising trend forecasted to continue through 2030 [2]. In 2019, HCC represents the fifth and seventh leading cause of cancer death in the U.S. men and women, respectively [3]. The aggressive clinical behavior and few effective therapeutic options are responsible for the overall high mortality of HCC.

Majority of HCC cases are considered potentially preventable because the major risk factors for HCC, including hepatitis B virus (HBV) and hepatitis C virus (HCV) infection, excess alcohol consumption, dietary aflatoxin exposure, smoking, and obesity, are generally modifiable. Over the past decades, considerable effects have been devoted to develop and improve prevention and surveillance for HCC at-risk populations. Neonatal HBV vaccination has been demonstrated to effectively reduce HBsAg seroprevalence in HBV-endemic countries and is now recommended in most countries [4, 5]. In the United States, chronic HCV infection confers the highest relative risk of HCC and accounts for approximately 24% of cases [6, 7]. One-time HCV screening in adults born between 1945 and 1965 and HCV antiviral therapies in high-risk individuals have been developed to combat HCV infection, but their benefits in real-world populations are a subject of debate, largely due to low attendance to screening and high proportions of asymptomatic HCV infection [8]. HCC surveillance using ultrasound (with and without alpha-fetoprotein) of patients with cirrhosis has been recommended by major liver disease societies of the world [9–11]. These recommendations are based on evidence from trials and observational studies indicating that HCC surveillance in high-risk individuals is associated with early diagnosis, mortality reduction, and prolonged survival [12–14]. However, conflicting evidence exists and concern over strength and inconsistency of these findings hinders acceptance of HCC surveillance by the National Cancer Institute, which concluded no mortality advantage of HCC routine screening but possible complications from screening [15, 16].

Aside from prevention and surveillance efforts, significant therapeutic advances in HCC have been made over the past decades, including increasingly available therapeutic options particularly effective systemic therapy, multidisciplinary clinical decision making, and development of prioritization criteria for liver transplantation [9]. These advances are thought to be significant in improving long-term response and survival of HCC patients, but whether and to what extent these therapeutic advances, along with surveillance efforts, have translated into clinically meaningful outcomes for the real-world populations are largely unclear.

The aim of our study was to investigate trends in patient characteristics and survival outcomes of HCC using the National Cancer Institute’s Surveillance, Epidemiology, and End Results (SEER) database, which collects data with longitudinal follow-ups from population-based cancer registries. We sought to explore prognostic factors influencing HCC survival among U.S. adults and determine whether year of initial diagnosis is independently associated with survival outcomes.

## Methods

### Study design and study population

A retrospective cohort analysis of HCC patients was performed using the SEER 18 registries database, which provides coverage of approximately 28% of the U.S. population [17]. Patients with HCC from 1988 to 2015 were identified using the International Classification of Disease for Oncology, Third Edition (ICD-O-3) histology codes 8170/3 and 8172/3–8175/3, with the liver site code C22.0. Fibrolamellar histology (8171/3) was excluded because of its distinct clinical features from conventional HCC [18]. Patients diagnosed before 1988 were excluded due to insufficient staging data. Additionally, patients were excluded if below the age of 18 years, not diagnosed as the first or only primary HCC, or diagnosed at autopsy or death certificate only. Individual patient data were retrieved using the SEER*Stat software version 8.3.5 [19]. This study was exempt from local research ethics committee approval considering that SEER data are de-identified and publicly available for research use.

Data pertaining to year of diagnosis, demographics (age at diagnosis, sex, race, and ethnicity), staging information (SEER summary stage, tumor extent, tumor size, and lymph nodes involvement), therapy, and survival follow-up (vital status, cause of death, and survival months) were included for analysis. To ensure consistency when comparing data over time, the SEER summary stage was used to classify stage at diagnosis, where localized, regional, and distant HCC are defined respectively as no evidence of extrahepatic spread, spread outside the primary organ to regional lymph nodes or tissues (along the hepatic pedicle, inferior vena cava, hepatic artery, porta hepatis, and periportal basins), and metastasis to distant lymph nodes or distant organs. Therapy received was categorized as no surgery performed, liver-directed therapy, hepatic resection/lobectomy, and hepatectomy with transplant. Liver-directed therapy includes photodynamic therapy, electrocautery, fulguration, cryosurgery, laser, alcohol ablation, heat radiofrequency ablation, and other local tumor destruction.

### Statistical analyses

Patient characteristics were summarized as mean ± standard deviation (SD) for numerical variables and as number and frequency for categorical variables. Year of initial diagnosis was grouped at 5-year interval and treated as an ordinal variable. Age 45 was used as the cut-off for defining young age-onset HCC and age was grouped accordingly into 10-years intervals. The Jonckheere-Terpstra test and Cochran-Mantel-Haenszel test were used to examine the trend association between period of diagnosis year and patient characteristics of numerical data and categorical data, respectively. Overall survival (OS), defined as the interval between initial diagnosis and death from any cause, was analyzed using the Kaplan-Meier method and difference between survival curves was evaluated using the log-rank test. Death within 30 days of initial diagnosis was defined as perioperative death and was recorded as 0 in survival time in SEER. For disease-specific survival (DSS) analysis, survival was censored at death from causes other than the primary disease. Survival rates at year 1, 3, and 5 were calculated for each period of diagnosis and stage. Cox proportional hazards regression models were used to obtain hazard ratios (HRs) and 95% confidence intervals (CIs) CIs for prognostic factors for OS and DSS. In the multivariate Cox regression analyses, a stepwise procedure (*P* < 0.15 for entry and *P* > 0.05 for removal) was employed to retain the most significant prognostic factors. Period of diagnosis was included in the final multivariate models as an independent variable of interest. The significance level was set at *P* < 0.05 in all statistical tests. Statistical analyses were performed with SAS 13.2 (SAS Institute, Cary, NC, USA). Kaplan-Meier curves were plotted using GraphPad Prism 7.0 (GraphPad Software, San Diego, CA, USA).

## Results

### Patient characteristics

**Fig. 1:**
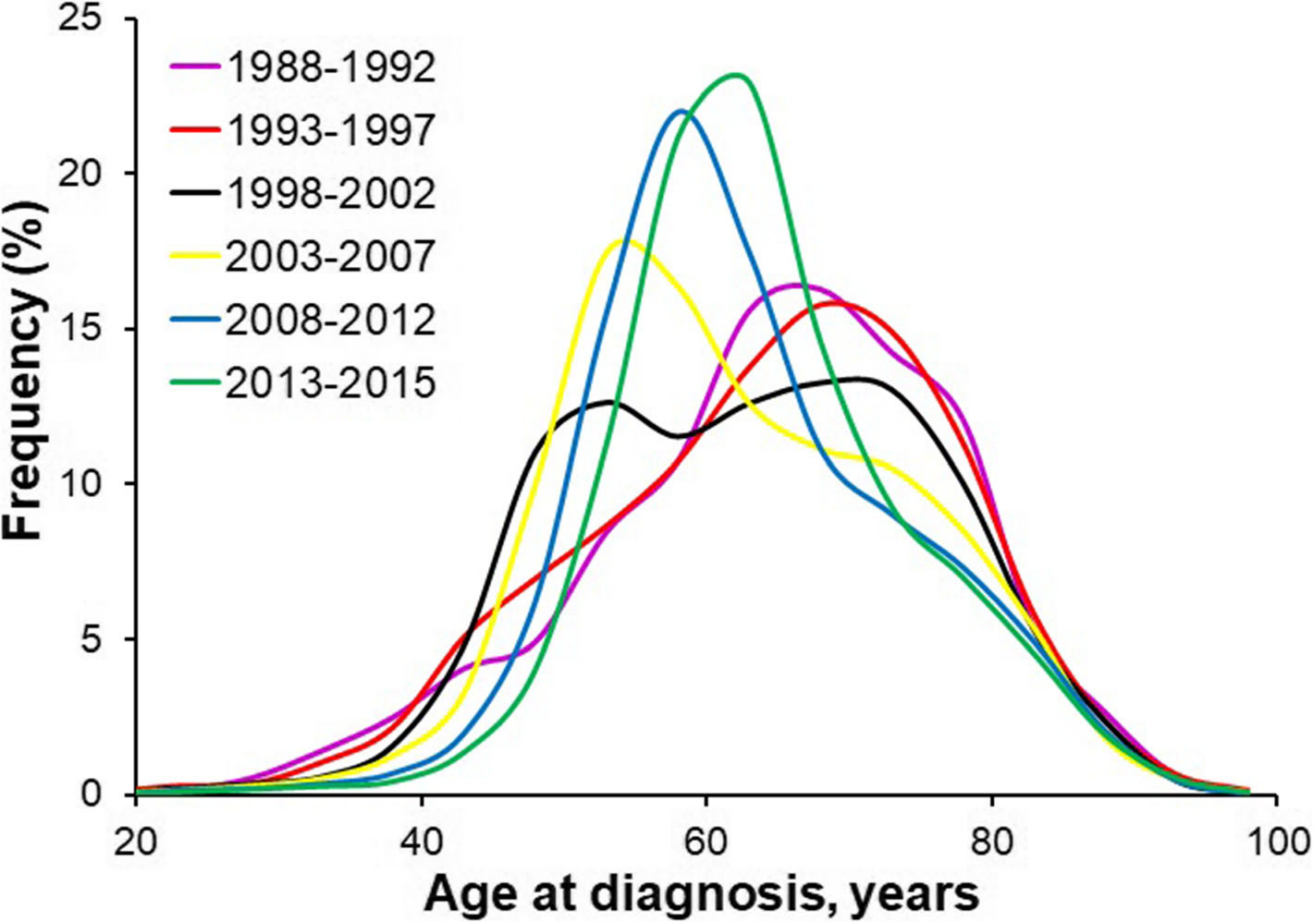
Age distribution at diagnosis of patients by period of diagnosis

The study cohort comprises 80,347 adult patients diagnosed with primary HCC between 1988 and 2015. Table 1 summarizes demographic, tumor, and therapy characteristics stratified by six time periods of initial diagnosis of HCC. The majority of HCC patients were male (76.6%), with the male-to-female ratio increasing from 2.70 of year 1988–1992 to 3.43 of year 2013–2015 (*P*_*trend*_ < 0.001). The average age at diagnosis was 62.0 years in male patients and 66.9 years in female patients, showing significant sex difference (*P* < 0.001). The proportions of both young patients (< 45 years) and old patients (≥75 years) decreased significantly over time. Figure 1 depicts the age distribution by period of diagnosis. Notably, age at diagnosis peaked at around 65–70 years for patients diagnosed during the first two periods (1988–1992 and 1993–1997), while for patients diagnosed after 2002, age at diagnosis peaked earlier at around 50–65 years. Whites (66.1%) and non-Hispanics (81.3%) made up the majority of HCC cases, while the composition of race and ethnicity in the cohort changed over time (*P*_*trend*_ < 0.001 for both).

**Table 1:** Characteristics of patients with hepatocellular carcinoma by period of diagnosis year

Year of diagnosis	Total	1988–1992 (***n*** = 2596)	1993–1997 (***n*** = 4992)	1998–2002 (***n*** = 10,737)	2003–2007 (***n*** = 18,387)	2008–2012 (***n*** = 25,500)	2013–2015 (***n*** = 18,138)	***P***_**trend**_
**Age (years)**^a^	63.1 ± 11.5	64.7 ± 12.8	64.4 ± 13.0	63.0 ± 12.8	62.2 ± 12.1	62.8 ± 11.0	63.9 ± 10.1	0.002
**Age in groups (years)**								0.233
18–44	3205 (4.0)	203 (7.8)	388 (7.8)	663 (6.2)	853 (4.6)	730 (2.9)	368 (2.0)	
45–54	15,033 (18.7)	324 (12.5)	754 (15.1)	2440 (22.7)	4518 (24.6)	4722 (18.5)	2275 (12.5)	
55–64	28,020 (34.9)	641 (24.7)	1158 (23.2)	2552 (23.8)	5562 (30.2)	10,337 (40.5)	7770 (42.8)	
65–74	19,640 (24.4)	812 (31.3)	1540 (30.8)	2845 (26.5)	4065 (22.1)	5504 (21.6)	4874 (26.9)	
≥ 75	14,449 (18.0)	616 (23.7)	1152 (23.1)	2237 (20.8)	3389 (18.4)	4207 (16.5)	2848 (15.7)	
**Sex**								< 0.001
Male	61,525 (76.6)	1895 (73.0)	3630 (72.7)	7973 (74.3)	14,113 (76.8)	19,869 (77.9)	14,045 (77.4)	
Female	18,822 (23.4)	701 (27.0)	1362 (27.3)	2764 (25.7)	4274 (23.2)	5631 (22.1)	4090 (22.6)	
**Race**								< 0.001
White	53,117 (66.1)	1546 (59.6)	2884 (57.8)	6828 (63.6)	12,117 (65.9)	17,267 (67.7)	12,475 (68.8)	
Black	10,526 (13.1)	333 (12.8)	546 (10.9)	1249 (11.6)	2338 (12.7)	3554 (13.9)	2506 (13.8)	
Other	16,406 (20.4)	713 (27.5)	1555 (31.1)	2628 (24.5)	3899 (21.2)	4587 (18.0)	3024 (16.7)	
**Ethnicity**								< 0.001
Hispanic	15,046 (18.7)	238 (9.2)	707 (14.2)	1810 (16.9)	3415 (18.6)	5036 (19.7)	3840 (21.2)	
Non-Hispanic	65,301 (81.3)	2358 (90.8)	4285 (85.8)	8927 (83.1)	14,972 (81.4)	20,464 (80.3)	14,295 (78.8)	
**Marital Status**^b^								< 0.001
Married	41,744 (54.4)	1643 (65.6)	2987 (61.4)	6071 (58.8)	9886 (55.7)	12,542 (51.9)	8615 (50.5)	
Not married	34,928 (45.6)	860 (34.4)	1874 (38.6)	4258 (41.2)	7865 (44.3)	11,634 (48.1)	8437 (49.5)	
Unknown	3675 (4.6)	93 (2.5)	131 (3.6)	408 (3.8)	636 (3.5)	1324 (5.2)	1083 (6.0)	
**SEER Stage**^b^								< 0.001
Localized	35,916 (50.8)	580 (32.5)	1500 (39.5)	4033 (45.3)	8244 (50.5)	12,346 (53.3)	9213 (55.1)	
Regional	22,235 (31.5)	611 (34.2)	1288 (33.9)	2881 (32.4)	5152 (31.5)	7177 (31.0)	5126 (30.6)	
Distant	12,538 (17.7)	596 (33.3)	1008 (26.6)	1988 (22.3)	2933 (18.0)	3630 (15.7)	2383 (14.3)	
Unstaged	9658 (12.2)	809 (31.2)	1196 (24.0)	1835 (17.1)	2058 (11.2)	2347 (9.2)	1413 (7.8)	
**Tumor Size (cm)**^b^								< 0.001
≤ 2	7393 (12.7)	68 (7.1)	134 (5.8)	601 (9.7)	1552 (11.8)	2744 (13.6)	2294 (15.0)	
2–5	23,919 (41.2)	284 (29.6)	732 (31.7)	2265 (36.7)	5246 (39.9)	8638 (42.8)	6754 (44.3)	
> 5	26,716 (46.0)	606 (63.3)	1444 (62.5)	3313 (53.6)	6352 (48.3)	8793 (53.6)	6208 (40.7)	
Unknown	22,319 (27.8)	1638 (63.1)	2682 (53.7)	4558 (42.4)	5237 (28.5)	5325 (20.9)	2879 (15.9)	
**Tumor Extent**^b^								< 0.001
Single lesion w/o vascular invasion	20,295 (34.7)	206 (19.8)	662 (26.7)	2040 (32.1)	4786 (34.1)	7240 (35.9)	5361 (37.2)	
Multiple lesions w/o vascular invasion	8597 (14.7)	76 (7.3)	236 (9.5)	697 (11.0)	2077 (14.8)	3169 (15.7)	2342 (16.2)	
Lesion(s) w/vascular invasion	29,572 (50.6)	757 (72.9)	1580 (63.8)	3613 (56.9)	7162 (51.1)	9739 (48.3)	6721 (46.6)	
Unknown	21,883 (27.2)	1557 (60.0)	2514 (50.4)	4387 (40.9)	4362 (23.7)	5352 (21.0)	3711 (20.5)	
**Lymph Node Involvement**^b^								< 0.001
Yes	5044 (8.6)	150 (20.8)	259 (15.8)	580 (11.6)	1107 (8.2)	1709 (7.9)	1239 (7.7)	
No	53,422 (91.4)	570 (79.2)	1383 (84.2)	4412 (88.4)	12,373 (91.8)	19,829 (92.1)	14,855 (92.3)	
Unknown	21,881 (27.3)	1876 (72.3)	3350 (67.1)	5745 (53.5)	4907 (26.7)	3962 (15.5)	2041 (11.3)	
**Therapy**^b^								0.001
No surgery performed	55,354 (77.0)			8645 (81.6)	13,285 (73.2)	19,573 (77.6)	13,851 (77.3)	
Liver-directed therapy	6764 (9.4)			579 (5.5)	1994 (11.0)	2273 (9.0)	1918 (10.7)	
hepatic resection/lobectomy	5638 (7.8)			828 (7.8)	1473 (8.1)	1972 (7.8)	1365 (7.6)	
Hepatectomy with transplant	4140 (5.8)			547 (5.2)	1394 (7.7)	1410 (5.6)	789 (4.4)	
Unknown	8451 (10.5)	2596^c^	4992^c^	138 (1.3)	241 (1.3)	272 (1.1)	212 (1.2)	

Data are *n* (%) unless otherwise specified

^a^ mean ± SD

^b^ unknown (missing) values were excluded from frequency distribution calculation and trend analysis

^c^ Surgery data were available since 1998

SEER summary stage, tumor size, tumor extent, and lymph node involvement were examined to evaluate temporal trends of disease severity at diagnosis. Overall, localized stage made up the largest proportion (50.8%) of HCC patients and this proportion increased gradually from 32.5% of year 1988–1992 to 55.1% of year 2013–2015 (*P*_*trend*_ < 0.001). Among patients with known tumor size, most patients diagnosed in 1988–1992 presented with primary tumor size > 5 cm (63.3%); this proportion decreased to 40.7% in 2013–2015. In contrast, tumor size ≤2 cm disease increased gradually from 7.1% in 1988–1992 to 15.0% in 2013–2015 (*P*_*trend*_ < 0.001). The proportion of vascular invasion diagnoses decreased significantly over time, from 72.9% of 1988–1992 to 46.6% of 2013–2015 (*P*_*trend*_ < 0.001). Lymph node involvement was reported in 7.7% of patients diagnosed in 2013–2015, significantly lower than that of patients in 1988–1992 (20.8%, *P*_*trend*_ < 0.001). These results should be cautiously interpreted given that a large proportion of cases diagnosed in early years were missing these disease severity data.

Detailed information regarding initial therapy received was available for patients diagnosed after 1998. As shown in Table 1, although the proportion decreased slightly over time, the majority of patients received no surgery (77.0%). The most common reason for no surgery was not recommended (90.1%). The proportions of patients underwent surgical resection and liver transplantation varied, but did not show significant trend over time. On contrast, the proportion of liver-directed therapy almost doubled from 5.5% in 1998–2002 to 11.0% in 2003–2007 and then leveled off afterward.

### Trends in survival

**Fig. 2:**
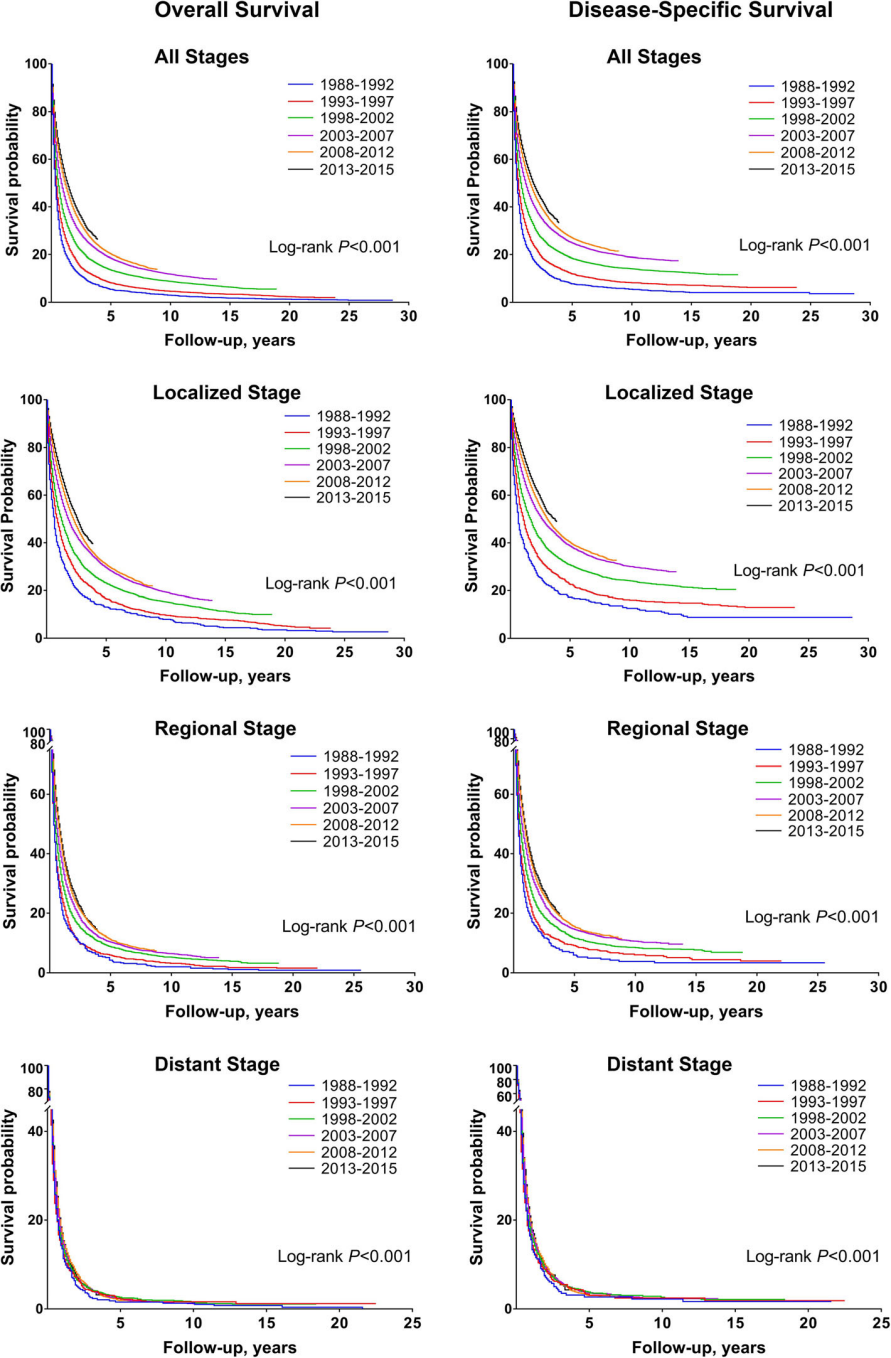
Kaplan-Meier survival curves for overall survival and disease-specific survival of patients stratified by period of diagnosis and SEER summary stage

OS and DSS after HCC diagnosis were 42.0 and 47.4% at year 1, 22.2 and 28.3% at year 3, and 15.6 and 21.5% at year 5, respectively, for the study cohort overall. Figure 2 shows Kaplan-Meier curves for OS (left panel) and DSS (right panel) stratified by period of diagnosis and disease stage. Significant improvement in OS and DSS regardless disease stage at diagnosis was seen over time (log-rank *P* < 0.001 for both). One-year OS rates increased steadily from 18.2 to 23.6%, 31.5, 40.2, 47.4, and 51.2%, respectively, over the continuously increasing periods of diagnosis. One-year DSS rates were 21.7, 28.5, 36.7, 45.9, 52.8, and 56.5%, respectively, over the 6 periods of diagnosis. Stratification by disease stage showed that the survival improvements over time were significant in all stage-stratified groups (log-rank *P* < 0.001), but were more pronounced in patients with localized and regional disease. In patients with localized HCC, one-year OS and DSS increased from 32.0 and 36.3% in 1988–1992 to 70.4 and 76.2% in 2013–2015, respectively. A large improvement in one-year survival was also observed for regional HCC (OS: 18.2 to 39.1%; DSS: 20.6 to 44.2%), and a modest improvement for distant HCC (OS: 10.0 to 14.2%; DSS: 13.2 to 18.1%). The unadjusted Cox model yielded a result consistent with the log-rank analysis and demonstrated a general decrease in HR with year of diagnosis (Table 2). Subsequent multivariate Cox analysis confirmed the independent prognostic significance of period of diagnosis, demonstrating a 25% reduction in the risk of cause specific death for patients diagnosed in 2013–2015, compared to those diagnosed in 1998–2002 (Table 3). Notably, subgroup analyses stratified by disease stage indicated that prognostic significance of period of diagnosis was evident in patients diagnosed with localized and regional HCC, not distant HCC.

**Table 2:** Univariate Cox regression analysis of demo-clinical characteristics predictive for survival outcomes of patients with hepatocellular carcinoma

	Overall Survival		Disease Specific Survival	
	HR (95% CI)	***P***-value	HR (95% CI)	***P***-value
**Year of diagnosis**		< 0.001*		< 0.001*
1988–1992	Ref		Ref	
1993–1997	0.89 (0.85–0.93)	< 0.001	0.86 (0.82–0.91)	< 0.001
1998–2002	0.71 (0.68–0.74)	< 0.001	0.68 (0.65–0.71)	< 0.001
2003–2007	0.59 (0.56–0.61)	< 0.001	0.55 (0.52–0.57)	< 0.001
2008–2012	0.52 (0.50–0.54)	< 0.001	0.48 (0.46–0.50)	< 0.001
2013–2015	0.46 (0.44–0.48)	< 0.001	0.42 (0.41–0.44)	< 0.001
**Age in groups (years)**		< 0.001*		< 0.001*
18–44	Ref		Ref	
45–54	1.05 (1.00–1.09)	0.03	0.99 (0.95–1.04)	0.75
55–64	1.03 (0.98–1.07)	0.23	0.97 (0.92–1.01)	0.11
65–74	1.19 (1.14–1.24)	< 0.001	1.13 (1.08–1.18)	< 0.001
≥ 75	1.60 (1.53–1.67)	< 0.001	1.50 (1.44–1.58)	< 0.001
**Sex**				
Female vs. male	0.94 (0.92–0.96)	< 0.001	0.94 (0.92–0.96)	< 0.001
**Race**				
White	Ref		Ref	
Black	1.14 (1.12–1.17)	< 0.001	1.14 (1.11–1.16)	< 0.001
Other	0.87 (0.85–0.89)	< 0.001	0.87 (0.85–0.89)	< 0.001
**Ethnicity**				
Hispanic vs. non-Hispanic	1.00 (0.98–1.02)	0.88	0.98 (0.96–1.00)	0.10
**Marital status**				
Married vs. not married	0.83 (0.82–0.84)	< 0.001	0.85 (0.83–0.86)	< 0.001
**SEER stage**		< 0.001*		< 0.001*
Localized	Ref		Ref	
Regional	1.98 (1.95–2.02)	< 0.001	2.18 (2,14–2.23)	< 0.001
Distant	3.58 (3.50–3.66)	< 0.001	4.08 (3.98–4.18)	< 0.001
**Tumor size (cm)**		< 0.001*		< 0.001*
≤ 2	Ref		Ref	
2–5	1.56 (1.51–1.61)	< 0.001	1.72 (1.65–1.79)	< 0.001
> 5	3.10 (3.00–3.20)	< 0.001	3.76 (3.62–3.91)	< 0.001
**Tumor extent**		< 0.001*		< 0.001*
Single lesion w/o vascular invasion	Ref		Ref	
Multiple lesions w/o vascular invasion	1.35 (1.31–1.39)	< 0.001	1.45 (1.40–1.50)	< 0.001
Lesion(s) w/vascular invasion	2.11 (2.06–2.15)	< 0.001	2.36 (2.31–2.42)	< 0.001
**Lymph node involvement**				
Yes vs. No	2.26 (2.19–2.33)	< 0.001	2.44 (2.36–2.52)	< 0.001
**Surgery**		< 0.001*		< 0.001*
No surgery performed	Ref		Ref	
Liver-directed therapy	0.38 (0.37–0.39)	< 0.001	0.34 (0.33–0.36)	< 0.001
hepatic resection/lobectomy	0.30 (0.29–0.32)	< 0.001	0.29 (0.28–0.30)	< 0.001
Hepatectomy with transplant	0.14 (0.14–0.15)	< 0.001	0.09 (0.09–0.10)	< 0.001

*Wald test

Abbreviation: HR, hazard ratio; CI, confidence interval

**Table 3:** Multivariate Cox regression analysis of demo-clinical characteristics predictive for disease specific survival of patients with hepatocellular carcinoma by stage

	Overall		Localized stage		Regional stage		Distant stage	
	HR (95% CI)	***P***-value	HR (95% CI)	***P***-value	HR (95% CI)	***P***-value	HR (95% CI)	***P***-value
**Year of diagnosis**								
1998–2002	Ref		Ref		Ref		Ref	
2003–2007	0.93 (0.88–0.98)	0.003	0.89 (0.83–0.96)	0.001	0.98 (0.91–1.07)	0.71	1.11 (0.93–1.33)	0.24
2008–2012	0.80 (0.76–0.84)	< 0.001	0.74 (0.69–0.79)	< 0.001	0.86 (0.79–0.93)	< 0.001	1.11 (0.93–1.32)	0.24
2013–2015	0.75 (0.71–0.79)	< 0.001	0.63 (0.59–0.68)	< 0.001	0.84 (0.77–0.91)	< 0.001	1.12 (0.94–1.34)	0.22
**Age in groups (years)**								
18–44	Ref		Ref		Ref		Ref	
45–54	1.17 (1.08–1.26)	< 0.001	1.31 (1.16–1.49)	< 0.001	1.07 (0.95–1.20)	0.29	1.11 (0.95–1.31)	0.19
55–64	1.14 (1.06–1.23)	< 0.001	1.31 (1.15–1.48)	< 0.001	1.01 (0.90–1.13)	0.91	1.08 (0.92–1.26)	0.35
65–74	1.27 (1.17–1.37)	< 0.001	1.56 (1.38–1.77)	< 0.001	1.07 (0.96–1.21)	0.24	1.09 (0.93–1.28)	0.27
≥ 75	1.53 (1.41–1.65)	< 0.001	1.96 (1.72–2.22)	< 0.001	1.25 (1.11–1.41)	< 0.001	1.19 (1.01–1.40)	0.04
**Sex**								
Female vs. male	0.94 (0.91–0.97)	< 0.001	0.93 (0.89–0.98)	0.002	*		*	
**Race**								
White	Ref		Ref		Ref		*	
Black	1.04 (0.99–1.08)	0.07	1.06 (1.00–1.13)	0.04	1.04 (0.98–1.10)	0.23	*	
Other	0.81 (0.78–0.84)	< 0.001	0.74 (0.70–0.77)	< 0.001	0.84 (0.79–0.89)	< 0.001	*	
**Ethnicity**								
Hispanic vs. non-Hispanic	0.96 (0.93–0.99)	0.02	*		0.93 (0.88–0.98)	0.009	*	
**Marital status**								
Married vs. not married	0.88 (0.86–0.91)	< 0.001	0.84 (0.81–0.87)	< 0.001	0.94 (0.90–0.98)	0.003	0.92 (0.86–0.97)	0.005
**SEER stage**								
Localized	Ref		*		*		*	
Regional	1.48 (1.43–1.55)	< 0.001	*		*		*	
Distant	2.54 (2.42–2.66)	< 0.001	*		*		*	
**Tumor size (cm)**								
≤ 2	Ref		Ref		Ref		Ref	
2–5	1.50 (1.42–1.57)	< 0.001	1.59 (1.49–1.69)	< 0.001	1.41 (1.28–1.55)	< 0.001	0.96 (0.83–1.11)	0.57
> 5	2.51 (2.39–2.64)	< 0.001	2.81 (2.64–3.00)	< 0.001	2.39 (2.18–2.62)	< 0.001	1.18 (1.03–1.35)	0.021
**Tumor extent**								
Single lesion w/o vascular invasion	Ref		Ref		Ref		Ref	
Multiple lesions w/o vascular invasion	1.36 (1.31–1.42)	< 0.001	1.44 (1.38–1.50)	< 0.001	1.13 (0.96–1.33)	0.15	1.09 (0.97–1.22)	0.17
Lesion(s) w/vascular invasion	1.31 (1.26–1.36)	< 0.001	1.27 (1.21–1.35)	< 0.001	1.42 (1.31–1.54)	< 0.001	1.23 (1.13–1.33)	< 0.001
**Lymph node involvement**								
Yes vs. No	1.14 (1.09–1.20)	< 0.001	*		1.30 (1.22–1.38)	< 0.001	*	
**Surgery**								
No surgery performed	Ref		Ref		Ref		Ref	
Liver-directed therapy	0.54 (0.52–0.56)	< 0.001	0.56 (0.53–0.59)	< 0.001	0.52 (0.48–0.56)	< 0.001	0.41 (0.33–0.51)	< 0.001
hepatic resection/lobectomy	0.35 (0.33–0.37)	< 0.001	0.34 (0.32–0.36)	< 0.001	0.38 (0.35–0.41)	< 0.001	0.35 (0.29–0.41)	< 0.001
Hepatectomy with transplant	0.14 (0.13–0.15)	< 0.001	0.14 (0.12–0.15)	< 0.001	0.14 (0.12–0.16)	< 0.001	0.33 (0.21–0.51)	< 0.001

*not included in multivariate model

Abbreviation: HR, hazard ratio; CI, confidence interval

### Univariate and multivariate analysis for survival

Table 2 shows the results of univariate analysis of survival outcomes stratified by demographic and clinical characteristics of the study cohort. The demographic predictors of worse survival were age ≥ 65 years at diagnosis, male, black race, and unmarried status. The “Other” race, defined as American Indian/Alaskan and Asian/Pacific Islander, mostly (93.8%) Asian/Pacific Islander, showed a significant survival advantage over white race. Advanced stage, increased tumor size, multiple lesions, vascular invasion, and lymph node involvement are disease-specific features that prognosticate worse survival. DSS in patients receiving no surgery, liver-directed therapy, surgical resection, and liver transplantation were 32.9, 79.8, 79.0, and 91.4% at year 1, and 13.2, 46.5, 57.1, and 79.6% at year 3, respectively. Compared with patients who had no surgery, patients who received liver-directed therapy, surgical resection, and liver transplantation achieved remarkably survival advantages (66, 71, and 91% reduction in risk of cause specific death, respectively).

Considering the magnitude of effect on survival associated with therapy and therapy data only available after 1998, we only included patients diagnosed after 1998 in the multivariate analysis. Subgroup analyses stratified by disease stage were also performed. As shown in Table 3, multivariate analysis confirmed the significance of age, sex, marital status as independent prognostic factors for HCC. Subgroup analyses revealed that the survival advantage of female over male was limited to patients with localized HCC. The “other” race retained significant survival advantage over white race in the adjusted Cox model, but prognostic benefit for whites over blacks was marginal. Hispanics had a slight survival advantage over non-Hispanics; subgroup analysis indicated that this prognostic effect of ethnicity was limited to patients with regional HCC after adjustment.

Tumor size, tumor numbers (multiple vs single), and vascular invasion were confirmed to be independent prognostic factors for HCC (Table 3). Subgroup analyses indicated strong prognostic powers of primary tumor > 5 cm and presence of vascular invasion in all stage-stratified groups. Lymph node involvement independently predicted increased risk of cause specific death (HR = 1.15, 95% CI: 1.10–1.20), but subgroup analysis indicated that its prognostic value was limited to regional disease.

HCC treatment, regardless therapy type, demonstrated significant survival advantages in the adjusted Cox model: there were 46, 65, and 86% reductions in risk of cause specific death for patients treated with liver-directed therapy, surgical resection, and transplantation, respectively. Subgroup analyses confirmed the strong survival impact of treatment across groups of patients diagnosed with localized, regional, and distant HCC. Hepatectomy with transplantation was associated with the greatest survival benefits across all stage groups: 86, 86, and 67% reductions of cause specific death risk were observed for patients with localized, regional, and distant HCC, respectively.

## Discussion

This study evaluated survival trend and determined prognostic factors affecting HCC survival in patients registered in the SEER 18 database of year 1988–2015. Our findings show that HCC survival has improved significantly over the study period, in parallel with a significantly increased proportion of patients diagnosed with localized disease and earlier peak of age distribution, likely reflecting improvements in early diagnosis in this real-world patient population. The period of initial diagnosis retained its independent significance in predicting survival on multivariate analysis and was significant in subgroup analyses in patients diagnosed with localized and regional disease, supporting that this temporal improvement in HCC survival is partially the results of therapeutic efforts, including and not limited to advance in imaging techniques [20], and emerging therapeutic options with curative intent [9].

The SEER data are broadly representative of the U.S. population and have high completeness and accuracy regarding disease diagnosis and follow-up, making observed trends in this study reliable and reflecting the real-world situation [21]. However, SEER database lacks information regarding detailed therapy activities and clinical factors such as performance status and liver function that affect risk stratification and treatment decision making; the correlation of these factors to survival outcomes is therefore not directly measurable. In this study, we used period of initial diagnosis as a surrogate for unmeasured and unknown factors that have offered increased survival advantage for HCC patients over time and our results clearly indicated survival advantages related to these unmeasured factors. HCC treatment paradigm has evolved over the past decades with the implementation of staging and scoring systems and advances in therapy modalities. The Barcelona Clinic Liver Cancer (BCLC) staging system, proposed in 1999, incorporates tumor stage with patient’s liver function and physical status to guide treatment schedule [22]. The BCLC staging is endorsed by American Association for the Study of Liver Diseases (AASLD) and is especially valuable to define early-stage patients who could benefit significantly from curative-intent therapies including percutaneous ablation, surgical resection and liver transplantation [9]. In the United States, the Milan criteria was introduced in 1996 to select HCC patients for liver transplantation and the Model for End-Stage Liver Disease (MELD) score was implemented in 2002 to prioritize liver transplantation candidates. This score system has benefited HCC patients because MELD exception points were granted to HCC patients based on the tumor burden, which resulted in a burst of liver transplants for HCC after 2002 [23]. Radiofrequency ablation (RFA) was approved by the U.S. Food and Drug Administration (FDA) in 2001 for general use in HCC patients and since then it is increasingly used [24]. RFA has achieved highly satisfactory results in treating early-stage HCC; outcomes of RFA as a first-line therapy were comparable with surgical resection, especially for single lesion ≤3 cm [25, 26]. Transarterial chemoembolization (TACE) is currently recommended as first-line therapy for patients with intermediate-stage HCC; the recommendation is based on prospective randomized trials published in 2002 showing survival benefit of TACE compared with the best supportive care or suboptimal therapies for patients with unresectable HCC [27]. TACE has also been widely practiced as bridging to liver transplantation by preventing tumor progression beyond the Milan criteria or even downstaging tumor into the Milan criteria for transplant [28]. Our findings of survival improvement in patients with localized and regional HCC support a meaningful positive influence of these treatment advances and efforts to the general population. The lack of survival improvement in patients with distant HCC is not unexpected and is likely explained by the fact that therapy modalities currently available for patients with advanced stage HCC are still limited. Sorafenib was approved in 2008 as the first chemotherapeutic therapy for advanced HCC, but its survival advantage seems to be restricted to patients with adequate liver functions and good performance status; its use has been limited to a small portion of patients [9]. The implementation of Sorafenib, along with other emerging chemotherapeutic agents, indicates a paradigm shift towards targeted systemic treatment, while their impact to HCC survival at population level remains to be unveiled.

We cannot rule out other possibilities such as changes in the etiology of HCC contributing to the survival improvement observed in this analysis. HCV-related HCC has shown the biggest proportional increases during the 1990s and early 2000, while nonalcoholic fatty liver disease (NAFLD) emerged as the fastest-growing etiology of HCC afterwards and is expected to suppress HCV as the leading indication for liver transplantation in the next few years in the United States [29, 30]. Patients with NAFLD-related HCC tend to present as a well-differentiated solitary lesion and have less severe liver dysfunction than those related to HCV and/or alcoholic liver disease, and therefore likely have better surgical outcomes and survival [31]. However, there are contradictory reports showing worse prognosis of NAFLD-related HCC related to advanced stage at presentation [32]. More studies are needed to assess the survival influence of increasing portion of NAFLD-related HCC cases at population level. The survival improvement of HCC was unlikely primarily related to HCC surveillance because adherence to HCC surveillance has remained low at < 20% of patients with cirrhosis and showed no obvious changes in surveillance uptake over the past two decades [33, 34].

We found HCC treatment, regardless type, was associated with a great survival advantage compared with patients receiving no surgery, which was independent of disease severity, period of diagnosis, and other factors. As expected, liver transplantation provided the most marked survival advantage than other therapeutic approaches. The survival benefit related to HCC treatment retained in subgroup analyses by disease stage, supporting a more aggressive treatment goal in HCC patients.

Survival analyses confirmed previous findings of prognostic significance of age, sex, marital status, tumor size, vascular invasion, and stage at diagnosis in HCC patients [35–39]. Controversial evidence exists regarding the independent significance of tumor size in predicting HCC survival [40, 41], and the optimal cut-off size seems ethnic specific and different cut-offs have been adopted by different staging systems [42]. The 8th AJCC TNM staging system subdivided HCC T1 category into T1a and T1b based on a tumor diameter cut-off of 2 cm and defined multiple lesions with the largest diameter being ≤5 cm as T2 [43]. In this study, we applied this cut-off and observed a significant worse prognosis in patients with primary tumor size > 5 cm regardless presence of vascular invasion, tumor number, stage at diagnosis, or surgery performed. The prognostic significance of tumor size > 5 cm was maintained in all stage-stratified groups, supporting incorporating tumor size as an independent factor into risk stratification in guiding treatment allocation.

A major limitation of this study is the limited availability of treatment regimens and some known prognostic factors for HCC in SEER data. For example, data regarding serum alpha-fetoprotein and liver fibrosis score, both demonstrating a good prognostic significance and commonly being considered in treatment decisions [44, 45], were not captured by SEER until 2004 and were therefore excluded from this study. Additionally, histological grade was not included in the multivariate analysis considering that 66.1% of patients were missing this data. Therefore, our study was unable to evaluate the contribution of these factors to the observed survival improvement in HCC patients. In addition, SEER registries do not provide information on the risk factors (such as HBV/HCV, alcohol use, smoking, obesity) or screening history, limiting the ability to assess relevant survival influence.

## Conclusions

Our results demonstrated that there has been a significant improvement in survival for HCC patients over the past three decades, which may be attributable to advances in early diagnosis and therapeutic approaches; however, patients diagnosed with distant HCC have not benefited substantially. Continued efforts to accelerate therapeutic advances in advanced HCC and improve the uptake of HBV vaccination and HCC surveillance may result in further survival improvement of HCC patients.

## Data Availability

The data used and/or analyzed during this study are available in an open database, the Surveillance, Epidemiology, and End Results (SEER) 18 Registries Data (https://seer.cancer.gov/).

## Abbreviations

HCC: Hepatocellular carcinoma
HBV: Hepatitis B virus
HCV: Hepatitis C virus
SEER: Surveillance, Epidemiology, and End Results
ICD-O-3: International Classification of Disease for Oncology, Third Edition
SD: Standard deviation
OS: Overall survival
DSS: Disease-specific survival
HRs: hazard ratios
CIs: Confidence intervals
BCLC: Barcelona Clinic Liver Cancer
AASLD: American Association for the Study of Liver Diseases
MELD: Model for End-Stage Liver Disease
RFA: Radiofrequency ablation
FDA: Food and Drug Administration
TACE: Transarterial chemoembolization
NAFLD: Nonalcoholic fatty liver disease
